# Comparison of Depth-Specific Prediction of Soil Properties: MIR vs. Vis-NIR Spectroscopy

**DOI:** 10.3390/s23135967

**Published:** 2023-06-27

**Authors:** Zhan Shi, Jianxin Yin, Baoguo Li, Fujun Sun, Tianyu Miao, Yan Cao, Zhou Shi, Songchao Chen, Bifeng Hu, Wenjun Ji

**Affiliations:** 1 College of Land Science and Technology, China Agricultural University, Beijing 100193, China; 2 State Key Laboratory of Remote Sensing Science, Aerospace Information Research Institute, Chinese Academy of Sciences, Beijing 100101, China; 3 Key Laboratory of Agricultural Land Quality, Ministry of Natural Resources, Beijing 100193, China; 4 College of Land and Environment, Shenyang Agricultural University, Shenyang 110866, China; 5 Institute of Applied Remote Sensing and Information Technology, Zhejiang University, Hangzhou 310058, China; 6 ZJU-Hangzhou Global Scientific and Technological Innovation Center, Hangzhou 311200, China; 7 Department of Land Resource Management, School of Public Finance and Public Administration, Jiangxi University of Finance and Economics, Nanchang 330013, China

**Keywords:** mid-infrared spectroscopy, visible and near-infrared spectroscopy, black soils, soil organic matter, soil total nitrogen, multivariate adaptive regression splines

## Abstract

The prediction of soil properties at different depths is an important research topic for promoting the conservation of black soils and the development of precision agriculture. Mid-infrared spectroscopy (MIR, 2500–25000 nm) has shown great potential in predicting soil properties. This study aimed to explore the ability of MIR to predict soil organic matter (OM) and total nitrogen (TN) at five different depths with the calibration from the whole depth (0–100 cm) or the shallow layers (0–40 cm) and compare its performance with visible and near-infrared spectroscopy (vis-NIR, 350–2500 nm). A total of 90 soil samples containing 450 subsamples (0–10 cm, 10–20 cm, 20–40 cm, 40–70 cm, and 70–100 cm depths) and their corresponding MIR and vis-NIR spectra were collected from a field of black soil in Northeast China. Multivariate adaptive regression splines (MARS) were used to build prediction models. The results showed that prediction models based on MIR (OM: RMSE_p_ = 1.07–3.82 g/kg, RPD = 1.10–5.80; TN: RMSE_p_ = 0.11–0.15 g/kg, RPD = 1.70–4.39) outperformed those based on vis-NIR (OM: RMSE_p_ = 1.75–8.95 g/kg, RPD = 0.50–3.61; TN: RMSE_p_ = 0.12–0.27 g/kg; RPD = 1.00–3.11) because of the higher number of characteristic bands. Prediction models based on the whole depth calibration (OM: RMSE_p_ = 1.09–2.97 g/kg, RPD = 2.13–5.80; TN: RMSE_p_ = 0.08–0.19 g/kg, RPD = 1.86–4.39) outperformed those based on the shallow layers (OM: RMSE_p_ = 1.07–8.95 g/kg, RPD = 0.50–3.93; TN: RMSE_p_ = 0.11–0.27 g/kg, RPD = 1.00–2.24) because the soil sample data of the whole depth had a larger and more representative sample size and a wider distribution. However, prediction models based on the whole depth calibration might provide lower accuracy in some shallow layers. Accordingly, it is suggested that the methods pertaining to soil property prediction based on the spectral library should be considered in future studies for an optimal approach to predicting soil properties at specific depths. This study verified the superiority of MIR for soil property prediction at specific depths and confirmed the advantage of modeling with the whole depth calibration, pointing out a possible optimal approach and providing a reference for predicting soil properties at specific depths.

## 1. Introduction

The northeast black soil region of China, as one of the three prominent black soil resource regions in the Northern Hemisphere, is an important grain production area [1,2]. However, soil degradation caused by intensive cultivation and soil erosion has been a serious problem, threatening the future sustainability of agriculture in the region [3]. Therefore, the rapid and accurate acquisition of spatial variability and physicochemical properties of information on field soil and the rational management of basic soil resources are important guarantees to meet the future population’s food and living needs and realize precision agriculture and sustainable agricultural development [4,5]. Soil not only exhibits physicochemical variations in horizontal directions but also in vertical directions, and vertical anisotropy of soil properties is frequently much stronger than lateral anisotropy [6]. The distribution of soil properties at different depths provides important reference information for soil classification and evolution. Obtaining soil properties at different depths is an important research topic for achieving three-dimensional visualization modeling of soil properties and promoting the development of precision agriculture.

Conventional analytical techniques, which develop valuable soil information over time, are expensive, laborious, and not eco-friendly [4,7]. As the demand for monitoring soil quality in agricultural production continues to increase, traditional soil chemical analysis methods are not able to meet the requirements for the rapid description of soil spatial distribution characteristics and changes, especially in large-scale studies. Therefore, the application of hyperspectral technology in the field of soil science has become increasingly widespread. Compared to traditional laboratory soil chemical analysis methods, spectroscopic techniques have the advantages of protecting soil integrity, characterizing multiple soil components, being cost-effective, reproducible, not requiring chemical reagents (environmentally friendly), and having simple preprocessing and higher efficiency when processing large numbers of samples [5,8,9,10]. In particular, mid-infrared spectroscopy (MIR, 2500–25,000 nm) contains more soil property information compared to visible and near-infrared spectroscopy (vis-NIR, 350–2500 nm) and has a huge potential for applications [11]. This is due to the fact that vis-NIR spectral bands represent internal octave and ensemble frequency vibrations of matter, resulting in significant overlap between information and difficulty in extraction, as noted in previous studies [12,13]. In contrast, MIR reflects the fundamental frequency of the internal molecular vibrations, enabling the identification of many soil properties that lack sensitive bands in the vis-NIR range. Thus, MIR has become increasingly popular in soil science research for its ability to capture a wider range of soil properties.

There have been some studies focusing on the prediction of soil properties based on spectra at multiple depths. Shahrayini et al. [14] evaluated the capability of vis-NIR to estimate soil organic carbon (OC) at multiple depths, including 0–15 cm, 15–40 cm, 40–60 cm, and 60–80 cm, confirming the capability of spectroscopy data in the range of vis-NIR to estimate OC concentration at multiple depths of the Doviraj plain in Iran. Xu et al. [15] evaluated the performance of vis-NIR to predict soil organic matter (OM), total nitrogen (TN), total phosphorus (TP), and total potassium (TK) at depths of 0 cm, 5 cm, 10 cm, 15 cm, and 20 cm and reported that vis-NIR combined with support vector machine regression (SVMR) has great potential to accurately determine the selected soil properties of intact soil cores of paddy fields. Viscarra Rossel et al. [16] successfully used proximally sensed vis-NIR to predict the content of soil clay at multiple depths including 0–10 cm, 10–20 cm, 20–30 cm, 30–40 cm, 40–60 cm, 60–80 cm, and 80–100 cm. The potential of the spectrum to predict soil properties (e.g., OC, OM, TN, TP, clay, silt, sand, etc.) at multiple depths (mostly among 0–100 cm) in in situ conditions has been reported by many researchers, along with some novel methods and devices [17,18,19,20,21,22]. However, despite the potential advantages of using MIR for predicting soil properties, most studies have focused on the application of vis-NIR to predict soil properties at different depths. Meanwhile, only a few researchers have tested the ability of a prediction model based on shallow layer calibration to predict soil properties at all depths and compared its prediction performances with the whole depth. If shallow calibration demonstrated acceptable performance, it would provide a more convenient and efficient alternative to collecting the deeper samples for calibration purposes.

This study explored the feasibility of using MIR for the prediction of soil OM and TN at different depths using whole depth and shallow layer calibration, respectively, to provide a reference for future research on soil property prediction at different depths. The study’s specific objectives included (1) analyzing the performance of MIR in predicting soil properties at different depths and comparing it with vis-NIR and (2) comparing the performance of using whole depth (0–100 cm) calibration with shallow layer (0–40 cm) calibration.

## 2. Materials and Methods

### 2.1. Study Area

**Figure 1:**
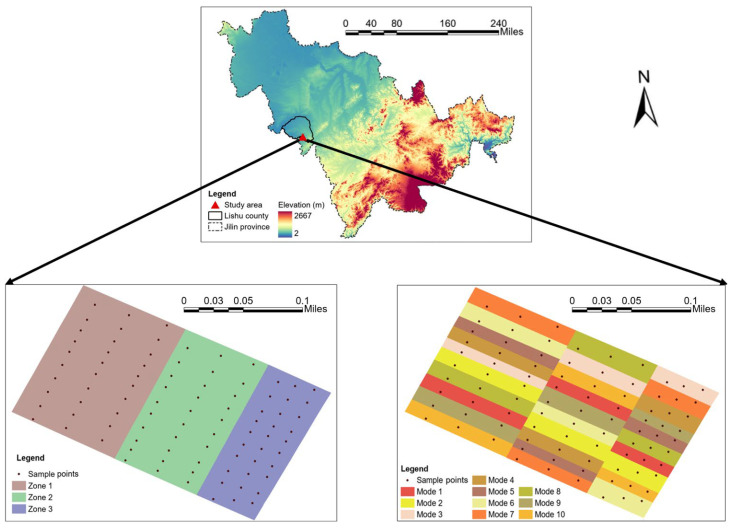
Study area and soil sampling points.

The study was conducted in an experimental field located in Lishu Country, Jilin Province, China (43°02′–43°46′ N, 123°45′–124°53′ E), as shown in Figure 1. The crop type was maize. The main soil type in the study area is black soil, classified as Phaoezem according to the World Reference Base for Soil Resources [23]. The climate is characterized as a semi-humid continental monsoon type with an average annual temperature of 6.4 °C and an average annual rainfall of 493 mm. The experimental field was divided into three parallel zones recorded as “Zone 1”, “Zone 2”, and “Zone 3”. Each zone was further divided into ten smaller areas based on different management practices recorded called “Ten Modes”.

### 2.2. Sample Collection

All soil samples in this study were collected in June 2020 with a hand soil auger. Soil samples were collected to a depth of 100 cm and divided into 5 intervals: 0–10 cm, 10–20 cm, 20–40 cm, 40–70 cm, and 70–100 cm. Overall, 3 soil samples were collected sequentially in each of the 3 zones and 10 modes of the “Ten Modes” experimental field, totaling 90 soil samples and 450 subsamples. The sampling point distribution and specific management practices of the “Ten modes” are shown in Figure 1 and Table 1. The management practices contained different tillage, spacing, and straw-returning methods. The spacing methods included “uniform spacing” and “narrow-wide” spacing. For example, “60 cm spacing” indicates that the crops were planted with a uniform spacing of 60 cm between each row. The “40:80 cm spacing” represents a narrow–wide row planting pattern. In this pattern, the spacing between rows alternates between a narrow distance of 40 cm and a wider distance of 80 cm. The wide row spacing allows for better airflow and light penetration, while the narrow row spacing optimizes resource utilization within each row. At the same time as collecting the soil samples, their coordinates were measured using a real-time kinematic (RTK) AgGPS 542 global navigation satellite system (GNSS) receiver (Trimble, Ltd., Sunnyvale, CA, USA). The coordinates corresponded to the point numbers and were placed in soil sampling bags with labels before being taken to the laboratory.

### 2.3. Spectral Measurement and Chemical Analysis

**Figure 2:**
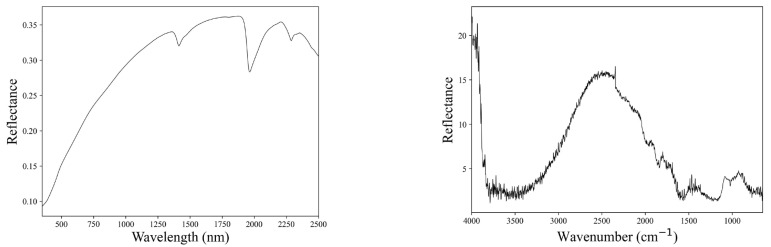
Vis-NIR spectrum (**left**) and MIR spectrum (**right**) of the same soil sample.

Soil samples underwent air drying, pulverization, and sieving via a 2 mm mesh screen. MIR spectral data were gathered using a 4300 handheld Fourier-transform infrared (FTIR) spectrometer (Agilent Technologies, Santa Clara, CA, USA) with a spectral range of 4000–650 cm^−1^ (2500–15380 nm), while vis-NIR spectral data were collected via a QualitySpec Trek portable spectrometer (Analytical Spectral Devices Inc., Boulder, CO, USA) with a spectral range of 350–2500 nm (Figure 2). MIR and vis-NIR spectra were collected from soils (more than 10 mm thick) and placed in plastic Petri dishes. Each sample was subjected to 3 iterative measurements. The mean of the replicate measurements served as the final spectral data for each sample. Spectrometers underwent whiteboard calibration every 10 min.

Laboratory chemical analysis indicators included soil organic matter (OM) and total nitrogen (TN). OM was calculated by multiplying the OC content determined by the FLASH2000 elemental analyzer (Thermo Fisher Scientific, Waltham, MA, USA) with a Van Bemmelen factor of 1.724 [24], and TN was also determined using a FLASH2000 elemental analyzer.

### 2.4. Spectral Pre-Processing

Spectral preprocessing plays a crucial role in enhancing the informative value of the spectral data. Frequently used methods include absorbance conversion (ABS) and Savitzky–Golay convolutional smoothing (SG) [25,26,27]. The parameters of SG smoothing include the polynomial order and the window size. Polynomial order controls the degree of fitting, while window size determines the number of data points used for smoothing. Larger window sizes and higher polynomial orders result in smoother outputs but may overfit the data. The ABS + SG (a polynomial of 2 with a window size of 15) combination was applied to the vis-NIR data, while the maximum normalization (MAN) +SG (a polynomial order of 2 with a window size of 11) combination was applied for the MIR data in this study after testing.

### 2.5. Predictive Algorithms

#### 2.5.1. Multivariate Adaptive Regression Splines

Friedman introduced the multivariate adaptive regression splines (MARS) algorithm in 1991 [28]. MARS is a highly flexible regression technique that is particularly well-suited for high-dimensional data analysis. Its main objective is to model complex relationships between response variables and predictor variables, making it a valuable tool for predicting soil properties.

The fundamental principle of MARS is the use of piecewise linear regression. It involves dividing the data space into smaller regions or segments and fitting linear functions within each segment. By allowing different segments to have their own unique linear models, MARS captures the nonlinear relationships present in the data. The partitioning of the data space is achieved through a process called forward selection and backward elimination. Starting with a single linear term, the algorithm progressively adds new terms or basis functions that improve the fit, while also considering their interactions with existing terms. At the same time, it selectively removes terms that do not contribute significantly to the model’s predictive power. This stepwise approach ensures that the model’s complexity is adjusted to the data, preventing overfitting. MARS also employs a regularization technique known as pruning to further enhance the model’s interpretability and generalization ability. Pruning involves iteratively simplifying the model by removing insignificant terms and reducing their impact on the final predictions.

In summary, MARS is a powerful and interpretable regression technique capable of capturing complex relationships in high-dimensional data. In this study, we utilized the MARS algorithm implemented in Python programming language (version 3.8) for data processing, analysis, and modeling.

#### 2.5.2. Data Splitting

In this study, 450 soil subsamples were collected from 90 soil samples from 5 different depths of 0–100 cm and divided into calibration and validation sets based on parallel zones. Specifically, soil samples from zones 1 and 2 were selected as the calibration set, consisting of 60 soil samples and 300 subsamples, while soil samples from zone 3 were used as the validation set, consisting of 30 soil samples and 150 subsamples.

### 2.6. Model Evaluation

In this study, the coefficient of determination (R^2^) and the performance of the model were evaluated using root-mean-square error (RMSE, RMSE_c_ for calibration, and RMSE_p_ for validation) and the ratio of standard deviation to the RMSE (RPD). The RMSE measures the difference between the predicted values and the actual values, while the RPD measures the quality of prediction relative to the variability of the measured data. The calculation formulas for the two metrics are as follows:

(1) $$R^{2}=1-\frac{\sum_{i=1}^{n}{\left(y_{\mathrm{obs}}-y_{\mathrm{pred}}\right)}^{2}}{\sum_{i=1}^{n}{\left(y_{\mathrm{obs}}-\;\bar{y}\right)}^{2}}$$

(2) $$\mathrm{RMSE}=\sqrt{1/n\overset{n}{\underset{i=1}{\sum }}{\left(y_{\mathrm{obs}}-y_{\mathrm{pred}}\right)}^{2}}$$

(3) $$\mathrm{RPD}=\mathrm{SD}/\mathrm{RMSE}={\left[\frac{1/\left(n-1\right)\sum_{i=1}^{n}\left(y_{\mathrm{pred}}-\;\bar{y}\right)}{1/n\sum_{i=1}^{n}\left(y_{\mathrm{obs}}-y_{\mathrm{pred}}\right)}\right]}^{1/2}$$

where n is the number of samples, $y_{\mathrm{pred}}$ is the predicted value, and $y_{\mathrm{obs}}$ is the actual value. SD is the standard deviation of the actual values, and RMSE is the root-mean-square error. A smaller RMSE indicates better agreement between the predicted and true values. A smaller RMSE indicates better agreement between the predicted and actual values, while a bigger RPD indicates better prediction performance relative to the variability of the measured data. When the RPD value is less than 1.4, the model is considered unreliable and lacks predictive ability. In the range of 1.4 to 2, the model is deemed reliable and exhibits good predictive ability. When the RPD value exceeds 2, the model is considered excellent, demonstrating outstanding predictive ability [29].

## 3. Results

### 3.1. Distribution of Soil Properties under Different Management Practices

**Figure 3:**
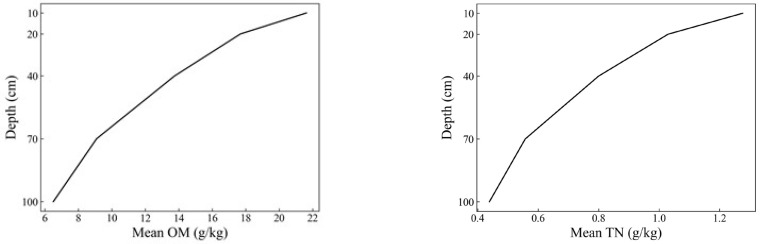
Mean OM (**left**) and TN (**right**) with soil depth.

**Figure A1:**
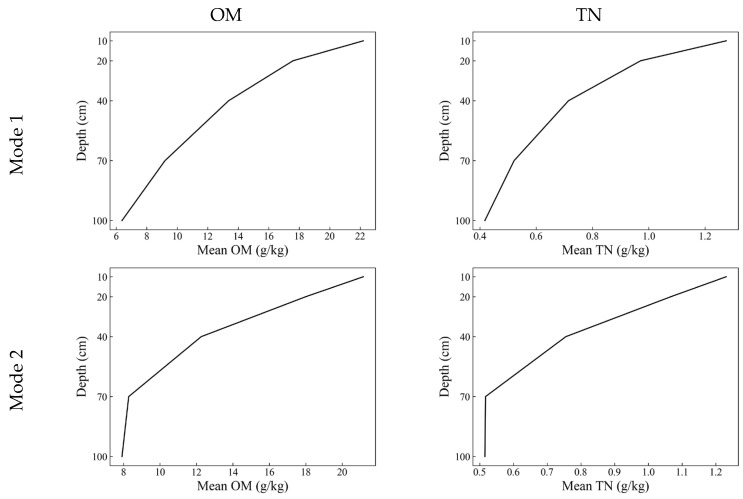

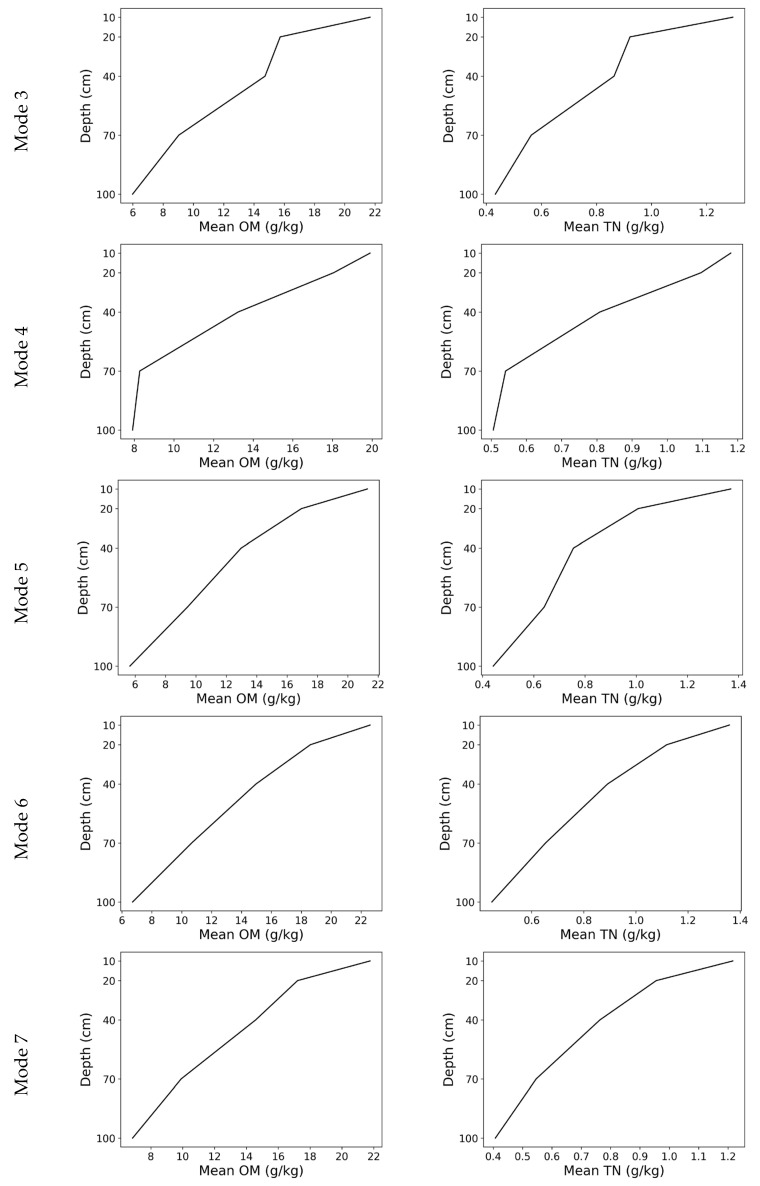

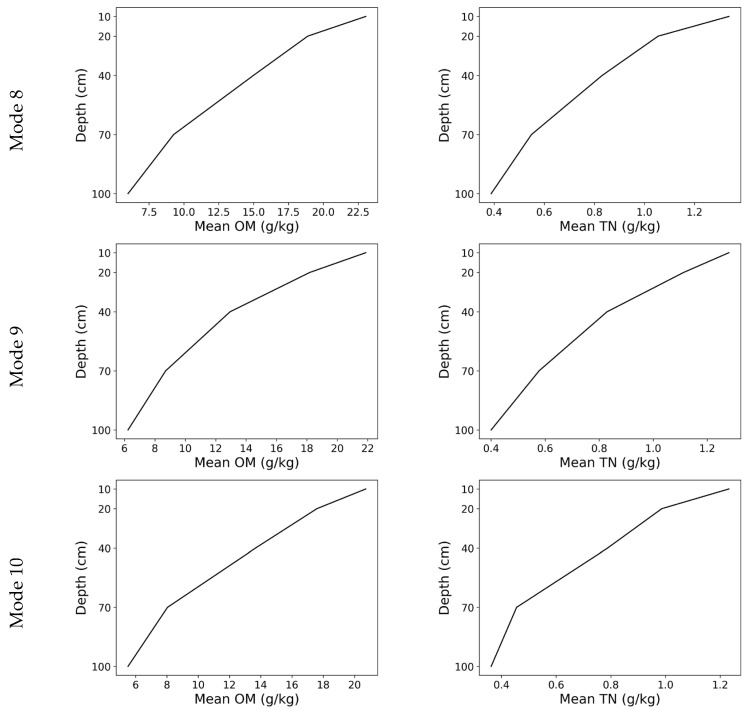
Change curves of soil properties with soil depth under ten different tillage modes.

The soil properties of both the whole depth (0–100 cm) and the shallow layers (0–40 cm) are presented in Table 2. For both OM and TN, the maximum values of the whole depth (0–100 cm) and shallow layers (0–40 cm) were equal. The mean and minimum values of the shallow layers (0–40 cm) were both greater than the whole depth (0–100 cm), while the standard deviation was smaller than the whole depth (0–100 cm). This indicated that the soil of the shallow layers had higher contents of OM and TN with lower variability. The variation between soil OM and TN contents with soil depth under ten different management practice modes is shown in Figure 3 and Figure A1, respectively. The content of soil OM and TN in the study area was mainly concentrated in the surface layer and rapidly decreased with depth. In terms of different management practices, mode 6 (no tillage, 40:80 cm spacing, straw returning) and mode 8 (no tillage, 40:140 cm spacing, straw returning) had relatively higher contents of OM and TN.

### 3.2. Prediction of Soil Properties at Different Depths with the Whole Depth (0–100 cm) Model

#### 3.2.1. Prediction of Soil Properties at Different Depths Based on MIR

**Figure 4:**
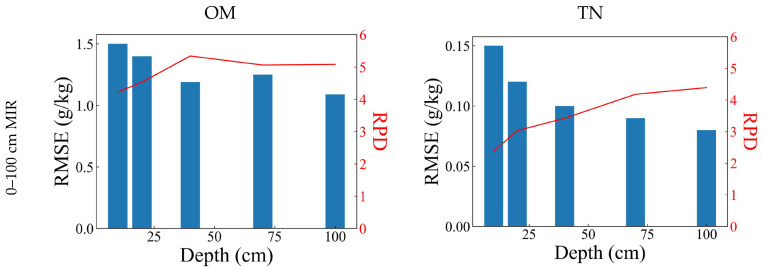

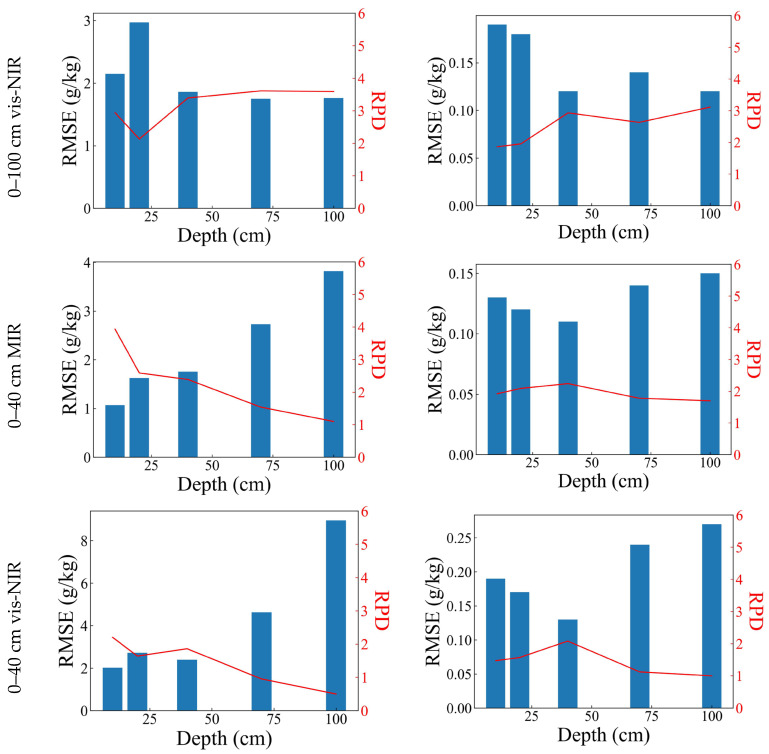
Prediction accuracy at different depths based on different spectra and calibrations.

**Figure 5:**
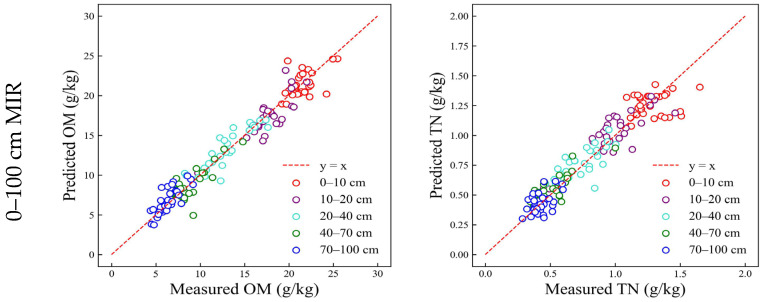

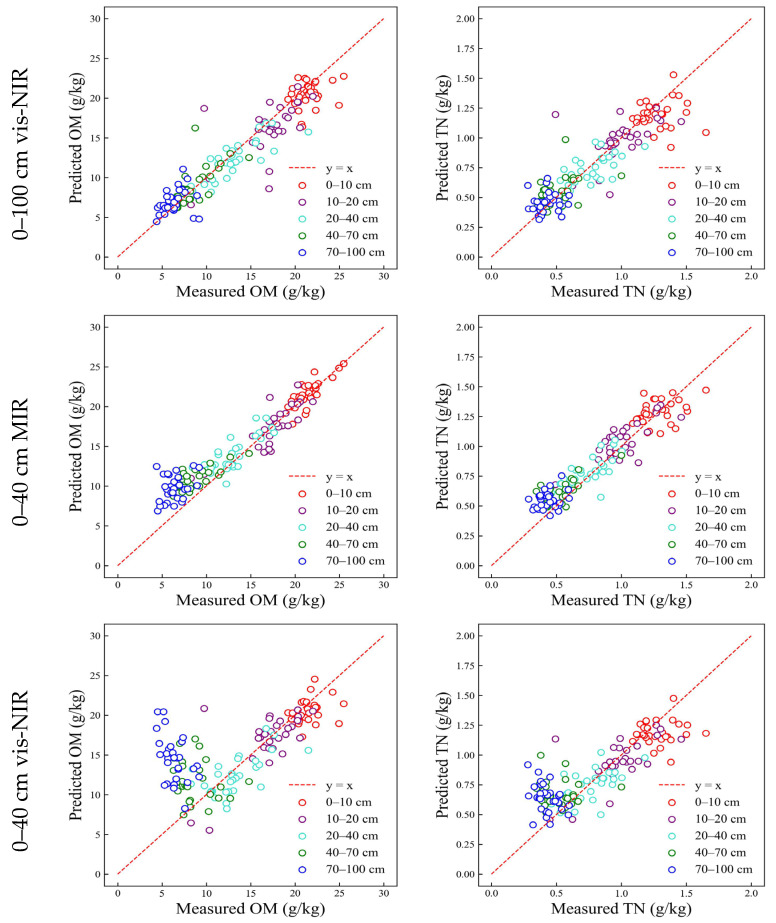
Prediction performance at different depths based on different spectra and calibrations.

The MIR-MARS model based on the whole depth (0–100 cm) could successfully predict OM (RMSE_c_ = 1.24 g/kg, RMSE_p_ = 1.09–1.50 g/kg, RPD = 4.22–5.80) and TN (RMSE_c_ = 0.11 g/kg, RMSE_p_ = 0.08–0.15 g/kg, RPD = 2.38–4.39), and the prediction performance varies at different soil depths (Figure 4 and Figure 5 and Table 3). For OM, the optimal prediction accuracy was found in 70–100 cm (RMSE_p_ = 1.09 g/kg, RPD = 5.80), while the performance was relatively poor in 0–10 cm (RMSE_p_ = 1.50 g/kg, RPD = 4.22) and 10–20 cm (RMSE_p_ = 1.40 g/kg, RPD = 4.53). For TN, the optimal performance accuracy was found in the 70–100 cm (RMSE_p_ = 0.08 g/kg, RPD = 4.39), while the performance was relatively poor in 0–10 cm (RMSE_p_ = 0.15 g/kg, RPD = 2.38).

The MIR-MARS model based on the whole depth exhibited good prediction performance for OM and TN.

#### 3.2.2. Prediction of Soil Properties at Different Depths Based on vis-NIR

The vis-NIR-MARS model based on the whole depth (0–100 cm) could successfully predict OM (RMSE_c_ = 2.48 g/kg, RMSE_p_ = 1.75–2.97 g/kg, RPD = 2.13–3.61) and TN (RMSE_c_ = 0.18 g/kg, RMSE_p_ = 0.12–0.19 g/kg, RPD = 1.86–3.11), and the prediction performance varies at different soil depths (Figure 4 and Figure 5 and Table 3). For OM, the optimal prediction accuracy was found in 40–70 cm (RMSE_p_ = 1.75 g/kg, RPD = 3.61) and 70–100 cm (RMSE_p_ = 1.76 g/kg, RPD = 3.59), while the performance was relatively poor in 10–20 cm (RMSE_p_ = 2.97 g/kg, RPD = 2.13). For TN, the optimal performance accuracy was found in the 70–100 cm (RMSE_p_ = 0.12 g/kg, RPD = 3.11), while the performance was relatively poor in 0–10 cm (RMSE_p_ = 0.19 g/kg, RPD = 1.86) and 10–20 cm (RMSE_p_ = 0.18 g/kg, RPD = 1.95).

The vis-NIR-MARS model based on the whole depth exhibited successful prediction performance for OM and TN. However, whether in the calibration set or any depth-specific validation set, the prediction accuracy of MIR was higher than vis-NIR, both for OM and TN.

### 3.3. Prediction of Soil Properties at Different Depths with the Shallow Layers (0–40 cm) Model

#### 3.3.1. Prediction of Soil Properties at Different Depths Based on MIR

For the depth-specific validation among the depths of shallow layers (0–10 cm, 10–20 cm, 20–40 cm), the MIR-MARS model based on the shallow layers (0–40 cm) could successfully predict OM (RMSE_c_ = 1.47 g/kg, RMSE_p_ = 1.07–1.76 g/kg, RPD = 1.10–3.93) and TN (RMSE_c_ = 0.13 g/kg, RMSE_p_ = 0.11–0.15 g/kg, RPD = 1.70–2.24), and the prediction performance varies at different soil depths (Figure 4 and Figure 5 and Table 3). For OM, the optimal prediction accuracy was found in 0–10 cm (RMSE_p_ = 1.07 g/kg, RPD = 3.93), while the performance is relatively poor in 20–40 cm (RMSE_p_ = 1.76 g/kg, RPD = 2.39). For TN, the optimal performance accuracy was found in the 20–40 cm region (RMSE_p_ = 0.11 g/kg, RPD = 2.24), while the performance was relatively poor in 0–10 cm (RMSE_p_ = 0.13 g/kg, RPD = 1.92). The prediction accuracy was generally lower than the MIR-MARS model based on the whole depth, which was higher only in at 0–10 cm.

The accuracy declined for the depth-specific validation of 40–70 cm (OM: RMSE_p_ = 2.73 g/kg, RPD = 1.54; TN: RMSE_p_ = 0.14 g/kg, RPD = 1.78) and 70–100 cm (OM: RMSE_p_ = 3.82 g/kg, RPD = 1.10; TN: RMSE_p_ = 0.15 g/kg, RPD = 1.70). The prediction for OM in 70–100 cm failed. However, the prediction accuracy for OM in 40–70 cm and TN in 40–70 cm and 70–100 cm was acceptable. This might be due to the relatively small scale and low soil variability.

In general, the MIR-MARS model based on the shallow layers exhibited lower prediction accuracy than the whole depth in the depth-specific prediction. Among the shallow layers, its depth-specific accuracy was still higher than the vis-NIR-MARS model based on the whole depth. In addition, it might have the potential to predict soil properties at deeper depths in some cases.

#### 3.3.2. Prediction of Soil Properties at Different Depths Based on vis-NIR

For the depth-specific validation among the depths of shallow layers (0–10 cm, 10–20 cm, 20–40 cm), the vis-NIR-MARS model based on the shallow layers (0–40 cm) could successfully predict OM (RMSE_c_ = 2.65 g/kg, RMSE_p_ = 2.02–2.72 g/kg, RPD = 1.64–2.20) and TN (RMSE_c_ = 0.17 g/kg, RMSE_p_ = 0.13–0.19 g/kg, RPD = 1.47–2.08), and the prediction performance varies at different soil depths (Figure 4 and Figure 5 and Table 3). For OM, the optimal prediction accuracy was found in 0–10 cm (RMSE_p_ = 2.02 g/kg, RPD = 2.20), while the performance was relatively poor in 10–20 cm (RMSE_p_ = 2.72 g/kg, RPD = 1.64). For TN, the optimal performance accuracy was found in the 20–40 cm (RMSE_p_ = 0.13 g/kg, RPD = 2.08), while the performance was relatively poor in 0–10 cm (RMSE_p_ = 0.19 g/kg, RPD = 1.47). The prediction accuracy was generally higher than the vis-NIR-MARS model based on the whole depth, which was lower only in 20–40 cm. This result was different from MIR-MARS, which indicated that it might not be helpful to take more data for calibration.

The accuracy obviously declined for the depth-specific validation of 40–70 cm (OM: RMSE_p_ = 4.63 g/kg, RPD = 0.96; TN: RMSE_p_ = 0.24 g/kg, RPD = 1.12) and 70–100 cm (OM: RMSE_p_ = 8.95 g/kg, RPD = 0.50; TN: RMSE_p_ = 0.27 g/kg, RPD = 1.00). All of them failed.

The vis-NIR-MARS model based on the shallow layers exhibited higher accuracy for depth-specific prediction among the shallow layers, but still had lower prediction accuracy than the MIR-MARS model. In addition, it could not predict the soil properties at deeper depths.

## 4. Discussion

### 4.1. The Connection between Soil Properties and Depth, as well as Management Practices

The soil’s organic matter and total nitrogen in the surface layer of the study area were highly concentrated and decreased rapidly with increasing depth. This might be due to the decrease in external input and the higher degree of organic matter mineralization in the deeper layers [30]. In addition, reducing tillage intensity was found to increase the abundance of aliphatic and phenolic functional groups in the upper soil layers, which were the primary drivers of soil organic matter (OM) accrual. Adopting a strategy of minimizing tillage intensity and rotating perennial forages in corn-based agricultural systems may result in a greater abundance of organic resources that drive OM accrual, potentially leading to additional benefits for soil health, plant roots, and soil organisms in conservative agricultural systems [31]. Jobbagy et al. [32] reported a study about the distribution of soil organic carbon based on three global databases of soil profiles, including the National Soil Characterization Database (NSCD), the World Inventory of Soil Emission Potential Database (WISE), and the Canadian Forest Service. They found that in the first meter of the soil profile (0–100 cm), the content of soil organic matter reduced as the soil depth increased and was significantly affected by plant functional types on it (such as grassland, shrubland, and forest). However, they did not consider agricultural soil because of the potential effects of plowing. Li et al. [20] analyzed the average distribution of organic carbon in the soil profiles of the three types of land use (arable, forest, and shrub meadow) and found that the concentration of organic carbon exhibits a gradual decline as the soil depth increases. At all depths, the mean carbon content in arable soil was found to be lower than forest and meadow soils. The organic carbon concentration in the top 40 cm of arable soil was substantially lower compared to forest soil. Moreover, the decline in OC concentration was more pronounced with an increase in soil depth. Xu et al. [15] and Wijewardane et al. [22] observed a decreasing trend of OM and TN along with an increase in depth. Zhang et al. [21] analyzed the distribution of soil organic matter at a depth of 0–100 cm and obtained a downward trend, which they attributed to the presence of sandy and clayey soils in the sub and deep soils.

### 4.2. Prediction Performance of Soil Properties with MIR vs. vis-NIR Spectra

The current study indicated that the prediction performance of soil OM and TN with MIR was greater than vis-NIR with either 0–100 cm or 0–40 cm calibration. The difference might be attributed to the fact that MIR spectra showed the fundamental frequencies of internal molecular vibrations, while vis-NIR spectra were related to the internal octave and ensemble frequency vibrations of materials, leading to a substantial overlap of information and challenges in extracting specific features. Consequently, many soil properties that lack sensitive bands within the vis-NIR range can be detected in the MIR range, thereby enhancing the predictive capacity of the respective models for soil properties at multiple depths [12,33]. Several previous studies have aimed to compare the capabilities of MIR and vis-NIR in predicting soil properties at various depths. For instance, Xie et al. [33] evaluated the performance of NIR and MIR for predicting soil organic carbon (SOC) and TN at different depths and found that MIR generally yielded better predictions. Numerous studies have further confirmed the effectiveness of MIR in predicting soil properties at different depths. Odgers et al. [34] developed continuous soil classes based on measured and MIR-predicted soil properties, while Chevallier et al. [35] successfully predicted various soil properties using MIR at depths ranging from 0 to 200 cm. Van de Broek et al. [36] demonstrated MIR as a reliable method for predicting SOC concentrations in tidal marsh sediments. Sanderman et al. [37] successfully predicted the distribution of SOC forms at depths up to 0–200 cm. These findings suggest that MIR holds significant potential for predicting soil properties at various depths and digital soil mapping. However, further research is needed to fully realize its capabilities.

### 4.3. Prediction Performance of Soil Properties with 0–100 cm vs. 0–40 cm Calibration

The current study showed that all models based on the whole depth (0–100 cm) and the shallow layers (0–40 cm) possessed the ability to accurately predict soil properties using corresponding validations. However, only the model based on the whole depth (0–100 cm) could steadily provide a successful prediction for all depth-specific validation. This was attributed to the larger sample size in the calibration and validation sets of the models, which was true for both the whole depth and shallow layer models. The soil sample data of the whole depth had a larger and more representative sample size and a wider distribution, aligning with the finding of Huang et al. [38], who showed that increasing sample size and representativeness was essential for enhancing model prediction performance. However, when it came to the prediction at different depths, the model established on 0–40 cm calibration could perform better. This might be due to the heterogeneous characteristics among the top and deeper soil sets. Some researchers [39,40] have reported that a spiking method [41] could be helpful by spiking some representative samples from the deep layers (0.1–0.3 m) into shallow layers. This method was trying to change the calibration to be more like the one based on the whole depth and also required the soil samples from the deepest layer, which could not significantly reduce the sampling workload.

In the present study, straw returning and conservation tillage measures caused the accumulation of organic matter and nitrogen in the surface layer, and they decreased rapidly with depth. Therefore, the models based on the shallow layers (0–40 cm) had difficulty learning the spectral characteristics when OM and TN contents were low, resulting in the overestimation of the prediction of deep soil properties. The model’s prediction outcomes for depths other than the corresponding training data were unreliable. Although the MIR-MARS model based on the shallow layers (0–40 cm) provided an acceptable prediction accuracy for OM in 40–70 cm and TN in 40–70 cm and 70–100 cm, it is more likely because of the relatively small scale and low soil variability. Further study needs to be carried out to confirm this.

### 4.4. A Potential Optimal Approach for Predicting Soil Properties at Specific Depths

This study verified that it was possible to predict the soil properties of a single soil layer at a specific depth based on spectra. It just needed a prediction model based on a calibration of the whole depth (in this study, it was all soil samples of 0–100 cm). On the one hand, this required a lower workload when researchers just need the properties at several specific depths, instead of the whole depth. On the other hand, this allowed researchers to predict soil properties at any depth with only one dataset, just like a little spectral library in the field scale. However, just like the spectral library, a global model often led to unsteady results [42,43,44,45] since the soil was a spatially inhomogeneous continuum with high spatial variability [46]. In this study, the model established on 0–40 cm calibration could provide higher accuracy for depth-specific prediction than the whole depth. Therefore, the theories and methods of soil property prediction based on the spectral library should be referred to in subsequent studies, such as locally weighted regression (LWR) [47], long short-term memory (LSTM) [48], etc., which might come up with a potential optimal approach for estimating soil properties at specific depths with lower sampling workload and higher accuracy.

## 5. Conclusions

This study verified the superiority of MIR spectroscopy for predicting soil properties at specific depths and highlighted the application potential of portable mid-infrared spectrometers. It also indicated that shallow layer calibration cannot accurately predict soil properties in deeper layers and confirmed the necessity of soil spectral libraries encompassing the entire depth range. Future efforts should prioritize the application of portable mid-infrared spectrometers and the development of more suitable algorithms for depth-specific prediction. Additionally, it is crucial to construct and utilize soil spectral libraries that incorporate spectra from multiple depths to enable the prediction of soil properties at various depths on a larger scale. Moreover, it is important to note that this study was conducted at the field scale. Therefore, future research should encompass a broader area characterized by greater soil variability to validate and generalize the findings obtained herein.

## Figures and Tables

**Table 1:** Management practices of the “Ten Modes”.

Mode	Management Practice		
Mode 1	ridge tillage	60 cm spacing	no straw returning
Mode 2	ridge tillage	60 cm spacing	straw returning (cover)
Mode 3	shallow rotary tillage	40:80 cm spacing	straw returning (after crushing)
Mode 4	deep rotary tillage	40:80 cm spacing	straw returning (after crushing)
Mode 5	rotary plowing	40:80 cm spacing	straw returning (after crushing)
Mode 6	no tillage	40:80 cm spacing	straw returning (cover)
Mode 7	no tillage	40:100 cm spacing	straw returning (cover)
Mode 8	no tillage	40:140 cm spacing	straw returning (cover)
Mode 9	strip tillage	40:90 cm spacing	straw returning (cover)
Mode 10	strip tillage	70 cm spacing	straw returning (cover)

**Table 2:** Statistical description of soil properties in the whole depth (0–100 cm) and shallow layers (0–40 cm).

Soil Properties	Whole Depth (0–100 cm)				Shallow Layers (0–40 cm)			
	Min.	Mean	Max.	SD	Min.	Mean	Max.	SD
OM (g/kg)	3.28	13.78	31.50	6.21	5.20	17.68	31.50	4.40
TN (g/kg)	0.22	0.82	1.86	0.36	0.35	1.03	1.86	0.28

**Table 3:** Prediction performance based on MIR and vis-NIR with 0–100 and 0–40 cm calibrations.

Calibration Type	Organic Matter (OM)					Total Nitrogen (TN)				
	Calibration		Validation			Calibration		Validation		
	R^2^	RMSE_c_	R^2^	RMSE_p_	RPD	R^2^	RMSE_c_	R^2^	RMSE_p_	RPD
0–100 cm MIR	0.96	1.24	0.95	1.29	4.90	0.90	0.11	0.90	0.11	3.23
0–100 cm vis-NIR	0.85	2.48	0.87	2.15	2.94	0.76	0.18	0.80	0.15	2.34
0–40 cm MIR	0.88	1.47	0.87	1.52	2.78	0.80	0.13	0.73	0.12	2.07
0–40 cm vis-NIR	0.65	2.65	0.68	2.40	1.86	0.62	0.17	0.64	0.17	1.65

## Data Availability

The data presented in this study are available upon request from the corresponding author.
